# Aurora at the pole and equator: overlapping functions of Aurora kinases in the mitotic spindle

**DOI:** 10.1098/rsob.120185

**Published:** 2013-03

**Authors:** Helfrid Hochegger, Nadia Hégarat, Jose B. Pereira-Leal

**Affiliations:** 1 Genome Damage and Stability Centre, University of Sussex, Falmer, Brighton BN21AU, UK; 2 Instituto Gulbenkian de Ciência, Rua da Quinta Grande, 6, Apartado 14, Oeiras 2781-901, Portugal

**Keywords:** Aurora kinases, microtubules, chromosome segregation, kinesin, Aurora evolution

## Abstract

The correct assembly and timely disassembly of the mitotic spindle is crucial for the propagation of the genome during cell division. Aurora kinases play a central role in orchestrating bipolar spindle establishment, chromosome alignment and segregation. In most eukaryotes, ranging from amoebas to humans, Aurora activity appears to be required both at the spindle pole and the kinetochore, and these activities are often split between two different Aurora paralogues, termed Aurora A and B. Polar and equatorial functions of Aurora kinases have generally been considered separately, with Aurora A being mostly involved in centrosome dynamics, whereas Aurora B coordinates kinetochore attachment and cytokinesis. However, double inactivation of both Aurora A and B results in a dramatic synergy that abolishes chromosome segregation. This suggests that these two activities jointly coordinate mitotic progression. Accordingly, recent evidence suggests that Aurora A and B work together in both spindle assembly in metaphase and disassembly in anaphase. Here, we provide an outlook on these shared functions of the Auroras, discuss the evolution of this family of mitotic kinases and speculate why Aurora kinase activity may be required at both ends of the spindle microtubules.

## 2. Introduction

**Figure 1.**
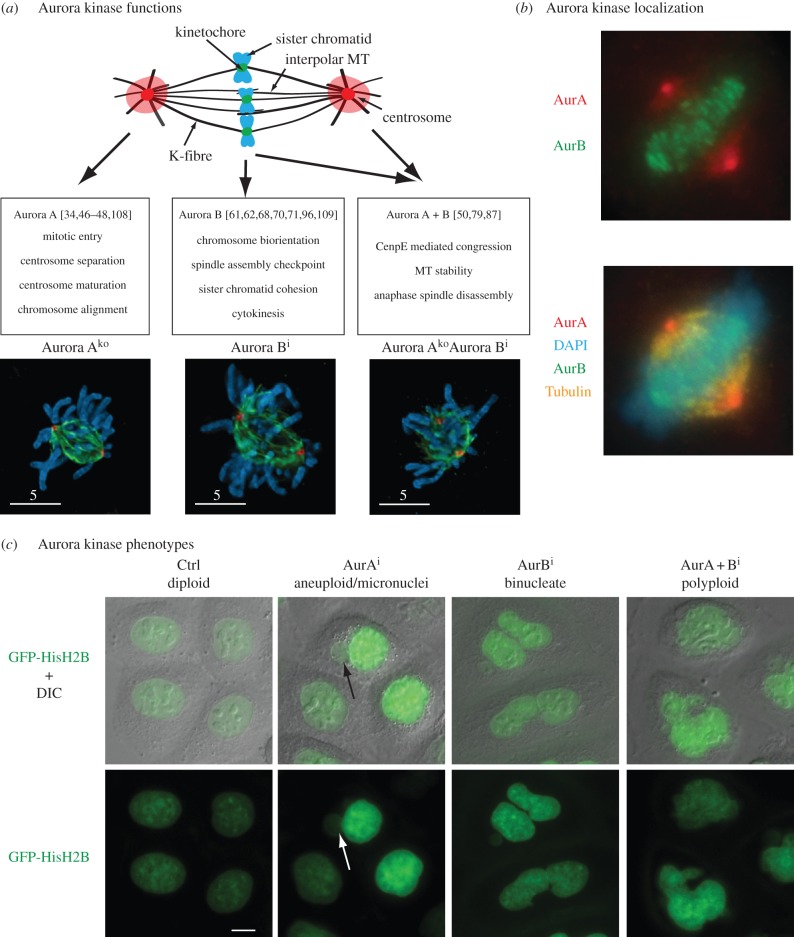
Specific and combined Aurora kinase functions. (*a*) Overview of functions of Aurora kinases in the mitotic spindle and images of cells lacking Aurora A, Aurora B and Aurora A+B activity [2]. (*b*) Centrosomal and centromeric localization of A and B-type Aurora in human *HeLa* cells. Immuno-fluorescent image of a formaldehyde fixed metaphase cell. (*c*) Nuclear phenotypes of *HeLa* cells expressing GFP-Histone H2B following 24 h incubation with Aurora A inhibitor (500 nM MLN8237), Aurora B inhibitor (60 nM AZD 1152) and Aurora A+B inhibitors (500 nM MLN8237+60 nM AZD1152). Aurora A inhibition causes micronuclei formation (see arrow) and aneuploidy [3,4], Aurora B inhibition results in a cytokinesis defect leading to binucleate cells [5,6], whereas inactivation of both Aurora A and B causes an abrogation in chromosome segregation and mitotic exit with a single quatroploid nucleus [2,7].

Each time a cell divides, it risks losing or gaining chromosomes. The resulting cellular aneuploidy can be detrimental and is a prominent cause of cancer formation [1]. The main task during mitosis is to ensure that the replicated sister chromatids are segregated with ultimate accuracy among the daughter cells. This is, in principle, a mechanical problem of generating force to segregate the two sister chromatids of each chromosome and move them to the opposite ends of the cell division plane. The mitotic spindle (figure 1*a*) provides the platform for accurate alignment of the condensed chromosomes and constitutes the molecular machine that segregates the sister chromatids [8,9]. It is crucial that the segregation process is only initiated when each chromosome is aligned in the centre of the spindle and bioriented, so that the sister chromatids in each chromosome are connected to opposite spindle poles. A complex signalling network that involves various checkpoints ensures this accurate timing [10–12]. Mitotic kinases constitute a key element of this regulatory network. More than 1000 proteins display mitosis-specific phosphorylation [13,14], and a growing number of kinases are implicated in executing these signalling events. Among them, Aurora kinases play a prominent role as essential regulators of the mitotic spindle and have been attributed a wide range of functions in mitotic control [15,16].

Glover and colleagues [17] discovered the first Aurora kinase in a screen for genes required to maintain the centrosome cycle in *Drosophila*. Mutant alleles in Aurora cause monopolar spindles [17]. A related budding yeast kinase, termed increased ploidy 1 (Ipl1) was later described to be required for chromosome segregation [18]. Orthologues of Aurora kinase were subsequently discovered in human cells [19,20] and in a variety of other model organisms [21–23]. It appears that in most unikonts Aurora kinase activity is required at both the spindle pole and the centromere. In higher eukaryotes, centrosome-associated Aurora kinases are now generally termed Aurora A, to distinguish them from the Ipl1-related Aurora B kinases that are a component of the chromosome passenger complex (CPC) [24]. A third mammalian Aurora paralogue, Aurora C [25], is functionally related to Aurora B [26,27] and thought to play a role in the meiotic cell cycle, but does not seem to be essential for cell divisions in somatic cells [28–30].

The Auroras are serine/threonine kinases with a highly conserved C-terminal kinase domain (Aurora A and B share 70% identity in their catalytic domain), but are found in separate protein complexes that determine their divergent localization and function. These distinctive interactions are mediated by a highly divergent N-terminus. The similarities between the paralogues are highlighted by the fact that a single amino acid change can turn Aurora A in a functional B-type Aurora that can replace endogenous Aurora B function [31,32]. Aurora A activation requires autophosphorylation of the activation loop [33,34], which is facilitated by forming a complex with the microtubule (MT)-binding protein Tpx2 [35,36] and counteracted by protein phosphatase 6 [37]. In addition, a variety of other proteins, such as Ajuba, Bora and Pak1 bind to and activate Aurora A at the centrosome [38–40]. Similar to Aurora A, activation of Aurora B requires autophosphorylation of the T-loop, which is, in this case, facilitated by the other members of the CPC, which are in turn also phosphorylated by Aurora B [41–45]. Inactivation of Aurora B is mediated by PP1 and PP2A phosphatases [46,47].

Thus, Aurora A and B interact with different sets of proteins, are differentially localized at the poles and the centromeres of the spindle, and are activated by separate mechanisms. These observations have led to the common notion that Aurora A and B function in unrelated and distinct aspects of mitotic control. Alternatively, these kinases could be required to work in the same signalling pathways from their respective residence at the opposite ends of the spindle MTs. Indeed, recent evidence suggests that Aurora A and B cooperate closely in regulating chromosome congression and alignment, metaphase spindle stability and anaphase MT dynamics.

This review aims to summarize the current knowledge on the concerted action of centrosomal and centromeric Aurora activity. Detailed overviews of individual Aurora A and B structure, functions, interactions and activation mechanisms have been given in excellent recent reviews [15,16,48,49]. Below, we will give a brief summary on the specific roles of these kinases, and then review recent evidence on functions and substrates shared between A and B-type Aurora kinases in more detail. Figure 1*a,b* gives a general overview of functions and localization of Aurora kinases.

## 3. Aurora A

The hallmark phenotype of Aurora A mutations in flies is a centrosome separation defect resulting in monopolar spindles [17]. This has led to the common notion that Aurora A is a major driver of centrosome separation. However, depletion or inactivation of Aurora A in mammalian cells only results in a modest increase in monopolar spindles [3,50], although other studies report more severe defects in centrosome separation after Aurora A inactivation using antibodies and in mouse embryonic fibroblast knockouts [51,52]. We recently reported that a conditional deletion of Aurora A in chicken DT40 cells causes chromosome alignment defects as well as a reduction in spindle MTs, but does not interfere with spindle bipolarity [2]. These differences between model systems could be reconciled by the recent descriptions of separate Plk1- and Cdk1-dependent control pathways for centrosome separation [53–55]. Aurora A may act together with Plk1, but seems to be dispensable for Cdk1-driven centrosome separation [2]. The impact of these pathways could vary among cell types and organisms, explaining the divergent extent of monopolar spindle phenotypes caused by Aurora A inactivation in different systems.

Another major mitotic defect in vertebrate cells lacking Aurora A activity is chromosome misalignment (figure 1*a*), resulting in defective chromosome segregation and aneuploidy. It is not self-evident how a centrosomal kinase coordinates the congression and segregation of chromosomes at the metaphase plate. The answer to this problem probably lies in functions of Aurora A in the control of spindle dynamics. This could be caused by defects in centrosome maturation leading to diminished mitotic MT polymerization. Aurora A has been shown to contribute to centrosome maturation in a variety of systems [38,56,57], and has been linked to this process via targets such as centrosomin and NDEL1 [58,59]. Aurora A also acts on other aspects of MT dynamics, and has been implicated in regulating a variety of MT-associated proteins that are involved in MT stabilization, destabilization and chromosome movement [50,60–63]. Most likely, the spindle defects and chromosome misalignment in Aurora A defective cells are the result of a complex interplay of various substrates. Functional relevance for most of these potential phosphorylation events is still lacking, and we are far from understanding the crosstalk between various Aurora A targets.

## 4. Aurora B

Aurora B is partnered with Incenp, Survivin and Borealin in the CPC, named after its transient localization to the chromosomes and inner centromere from pro- to metaphase, the central spindle in anaphase and the cleavage furrow during cytokinesis [24,64]. A critical function of Aurora B and the CPC is the control of chromosome biorientation [65]. The kinase destabilizes incorrectly attached MT–kinetochore connections via the MT deploymerase mitotic centromere-associated kinesin (MCAK) [66,67], and by targeting kinetochore components in the KNL1/Mis12/Ndc80 network and the Ska complex [68,69]. These phosphorylations are removed by PP1, once tension is established and the outer kinetochore is separated from the centromeric Aurora B [70–72]. By generating unattached kinetochores during error correction, Aurora B intrinsically impacts on the spindle assembly checkpoint (SAC), but a more direct involvement of Aurora B in the SAC has also been proposed [73]. Thus, Aurora B inhibition does affect SAC maintenance in response to loss of attachment [5,6] or when the SAC is caused by constitutive tethering of Mad1 to the kinetochore [74]. This could be explained by a possible role for Aurora B to recruit SAC components such as Mad2 and BubR1 to the kinetochore [6]. Furthermore, Aurora B plays a role in sister chromatid cohesion [75,76], spindle disassembly [77] and cytokinesis [78], and has meiosis-specific functions in regulating the synaptonemal complex [79]. These various roles reflect the differential localization of Aurora B at the chromosome arms, centromeres, central spindle and midbody during mitotic progression [80]. Cell division in the absence of Aurora B activity results in chromosome missegregation. However, the major consequence of Aurora B inhibition in mammalian cells is a cytokinesis failure resulting in binucleate daughter cells [64,81–83]. This suggests that Aurora B is not strictly essential for chromosome segregation and that the dominant Aurora B phenotype lies with its telophase functions in controlling abscission. As for Aurora A, the major task ahead in studying Aurora B lies in cataloguing and characterizing the functions of Aurora B-dependent phosphorylation of its various substrates.

## 5. Overlapping roles of centromeric and centrosomal aurora kinases

In summary, the major consequence of Aurora A inactivation in mammalian cells appears to be spindle pole separation defects, chromosome alignment defects and aneuploidy. Aurora B inhibition results in chromosome missegregation, cytokinesis defects and binucleated cells. Does this mean that these kinases work separately in unrelated compartments of mitotic control, or are there areas where these kinases work together, or have overlapping functions? What is the actual phenotypic consequence of combined inactivation of Aurora A and B? If these kinases were to have entirely non-related functions, one would expect a combination of chromosome segregation defects and the dominant Aurora B cytokinesis failure giving rise to binucleated cells. This would imply that defects in Aurora A and B signalling are completely separate without impact on each other. Given that these kinases play an important role in chromosome alignment and segregation, this is unlikely. Accordingly, we have recently demonstrated that inactivation of both Aurora A and B in chicken DT40 cells causes a much more severe defect in sister chromatid segregation, resulting in mitotic exit with a single tetraploid nucleus [2]. The same appears to be true if Aurora A and B are concomitantly inactivated in human cells [7] (figure 1*c*). This segregation failure could be caused by mitotic slippage owing to a SAC defect in response to Aurora B inactivation. This is, however, unlikely because SAC inactivation in Aurora A defective cells by other means (Mps1 inhibition, Mad2 depletion) does not interfere with chromosome segregation [2].

A complete failure in chromosome segregation could be the consequence of persistent sister chromatid cohesion, defective force generation in the mitotic spindle or a synergistic failure in chromosome congression. Aurora B is involved in the control of sister chromatid cohesion, but does not play an essential role in removing cohesin from the centromeres prior to anaphase [75,76]. To our knowledge, there is no evidence that Aurora A plays a role in cohesion. It does not act in the vicinity of the metaphase chromosomes and is unlikely to further contribute to the removal of cohesins during anaphase. Thus, it is more likely that failure of sister chromatid segregation in the absence of Aurora A and B kinase activity is an additive effect in the control of chromosome alignment, or MT dynamics that results in a failure to pull the sister chromatids apart.

How could A- and B-type Aurora kinases act together in the control of chromosome segregation? There could be cases of substrate redundancy, if Aurora A and B share a common substrate that both can phosphorylate. Alternatively, the observed additive effect could be the result of pathway redundancy, where separate centrosomal and centromeric Aurora substrates work in parallel at their respective ends. Proteomic data are available to compare specific and overlapping substrates of Aurora kinases [84]. The majority of Aurora substrates in this study appeared to be highly specific to either Aurora A or B, matching also in their respective localization to the centrosome and centromere. However, a number of proteins were clearly phosphorylated by both kinases. These double targets were mostly localized on the mitotic spindle, where they could meet either kinase from the centrosomal or centromeric ends. Recent studies have started to shed light on common substrates and functions of Aurora A and B kinases, and point to a complex interplay between the centromeric and centrosomal Aurora activity in coordinating mitotic spindle function. An overview of shared Aurora A and B targets is given in table 1.

**Table 1.** Overlapping substrates of Aurora A and Aurora B [84]. Italic, different sites phosphorylated by either Aurora A (MLN1+MLN5) or Aurora B (MLN5+AZDZM). Bold, same sites phosphorylated by Aurora A and B (inhibited by MNL1 and AZDZM). Remaining names, sites phosphorylated by either Aurora A or Aurora B (inhibition by MLN5).

categories	gene names
cytoskeleton-associated processes	
spindle organization/orientation	HAUS6, HAUS8, KIF23, SPAG5, *GPSM1, RANBP2*, TPX2, PARD3^a^, TCOF1, KIF4A, DLGAP5, NUMA1
centrosome cycle	NPM1, CC2D1A, OFD1
attachment of spindle microtubules to kinetochore	CASC5, SPAG5, CENPF, *CENPE* [92]
microtubule polymerization or depolymerization	ARHGEF2, SLAIN2, MAPRE3^a^, KIF18B, CEP170, KIF2A [63,138], *KIF2C* [95]
actin filament organization/ actin-associated proteins	ARHGEF2, LATS1, **ZYX**, PDLIM5, STK10, SSFA2, BAG3, ARHGEF18, DIAPH3, MYO9B, ABLIM3, PALLD, PLA2G4A, CAMSAP1
other microtubule-based processes	**MACF1**, **SPAST**, **GPHN**, NEK4, MAP7, ATL1, CLIP2
other microtubule-associated proteins with unknown functions	KLC1, MAP4, ASPM, *MAP1B*, MAP7D3, KLC2, MAP7D1, MAP7D2
present at centrosome but uncharacterized proteins/unknown functions	SYTL4, **PRKAR2A**, CEP170, WDR62
other cytoskeleton proteins/associated processes	**KRT17**, LMNA, LMNB1, SYNC, PLEC
DNA-associated processes	
other kinetochore/centromere proteins	**CENPV**, CENPC1, DSN1
chromosome condensation/sister chromatin cohesion	ACIN1, CDCA5, NCAPD2, NCAPG, NCAPH, TOP2A, GSG2, PDS5B, KIF4A
nucleosome organization	BAZ1B, HJURP, NPM1, *HIST1H3A*^a^
response to DNA damage stimulus	ATR, BAZ1B, CDCA5, NPM1, RASSF1^a^, SETMAR, TERF2IP, TP53^a^, TRIP12, UBR5
regulation of gene expression	ARHGEF2, CENPF, DAXX, MAP3K2, PSIP1, RBM14, RBMX, RNF25, RTF1, **PPP1R8, SUPT6H, RBM17, LMO7**, TAF15, ZFHX3, GTF2I, RREB1, KDM3B, RDBP
present on chromosomes with other functions	CDCA2, TMPO, BAZ1A
present on chromosomes with unknown functions	**MKI67**, MKI67IP
other processes	
G2/M transition of mitotic cell cycle	CENPF, LATS1, NES, CDC25B, MELK
regulation of cytokinesis	CDC25B, **CENPV**, KIF23, PRPF40A, **SPAST**, **ATXN10**, KIF4A, DIAPH3
others functions	TBC1D4, ARFGAP3, RCHY1, WWC1^a^, **RTKN, GRWD1**, EIF4ENIF1, RFC1, ANAPC7, AHNAK, CAD, NT5C2, PFKFB2, CDK16, PI4KB, AP4B1, NUP50, SIK1, DENND4C
uncharacterized proteins	**LUZP1**, NUCKS1, KCMF1, PDXDC1, CCDC86, TBC1D12, BOD1L, ZC3H11A, CDK17, MINA, MCTP2, CDK18, RPRD2, LIMCH1, C17orf59

^a^From phosphositeplus.org [139].

### 5.1. Coordinated action of Aurora A and B in the control of CenpE

**Figure 2.**
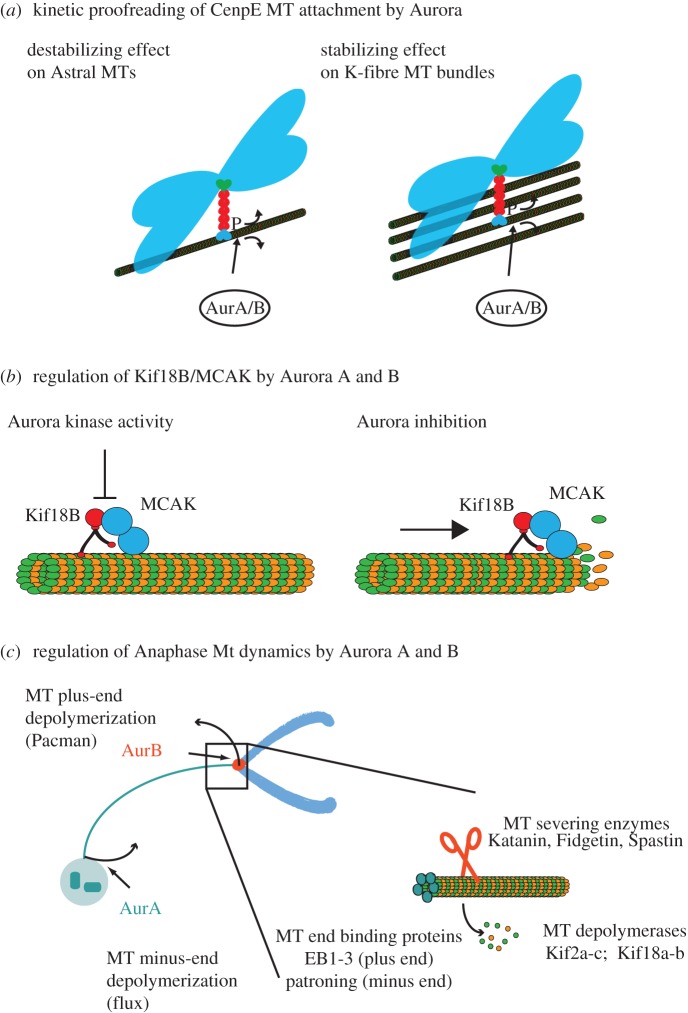
Aurora A and B kinase synergies. (*a*) CenpE phosphorylation by Aurora kinases results in kinetic proofreading **(**model adapted from Kim *et al.* [92]) Aurora kinase phosphorylation destabilizes CenpE binding to MTs. The protein is morel likely to re-attach to a neighbouring MT in the dense K-fibre bundles than on single astral MTs. (*b*) Control of Kif18b/MCAK interaction by Aurora- kinases (model adapted from Tanenbaum *et al.* [95]). The plus-ended motor Kif18b transports MCAK to the MT tip, where it depolymerizes the tubulin polymers. Aurora kinases jointly negatively regulate this interaction by an unknown mechanism. (*c*) Aurora controlled MT depolymerization in anaphase (model adapted from Hegarat *et al.* [2]). In metaphase, the kinetochores are attached to kinetochore MT fibres (K-fibres) that reach to the spindle poles. Minus-end depolymerization causes a constant flux of tubulin towards the spindle poles that is counteracted by plus-end MT polymerization at the kinetochore to achieve constant spindle length. In anaphase K-fibres are rapidly depolymerized at both plus and minus ends. This MT depolymerization releases energy that is used to pull the kinetochores along the shrinking K-fibre. Possible targets in this pathway are proteins that regulate MT stability. Among these, the end-binding proteins have been shown to be Ipl1 substrates in yeast, and this phosphorylation is linked to spindle disassembly [96]. MT-severing enzymes and MT depolymerases have also been shown to be targeted by Aurora kinases, but these phosphorylations are thought to negatively regulate their enzymatic activities [66,67,97].

One prominent mitotic player that is a target of both Aurora A and B is the plus-end-directed motor protein CenpE [85]. This kinesin is required for congression of chromosomes from the spindle poles to the equator [86,87]. MTs emanating from the spindle poles often capture chromosomes by lateral attachment of the kinetochore to the MT surface [88]. These captured chromosomes are transported towards the spindle pole by the minus-end-directed motor protein dynein [89–91]. In this way, kinetochores are exposed to an MT-dense area in vicinity of the spindle pole, increasing the chance of efficient MT attachment. CenpE is essential for transporting these polar chromosomes to the spindle equator, and removal of CenpE does result in a chromosome alignment failure, with chromosomes remaining at the spindle poles [92–94]. Given its function as a transporter of chromosomes from the pole to the equator, it makes sense that this protein is exposed to both Aurora A at the centrosome and Aurora B at the kinetochore. In an elegant study, Kim *et al.* [85] mapped an essential Aurora phosphorylation site in CenpE at Thr422, downstream of the coiled-coil neck that follows the kinesin motor domain. This site is indeed targeted by both Aurora A and B, and is essential for efficient chromosome congression. The authors demonstrated that Thr422 phosphorylation decreases the affinity of CenpE to MTs and reduces the motor's processivity. The site is also located within a docking motive for PP1 and opposes PP1 binding. This suggests a model whereby Aurora A phosphorylates CenpE on laterally attached chromosomes that have been transported to the spindle pole by dynein. The destabilizing effect of the Thr422 phosphorylation would inhibit tethering of CenpE to individual astral MTs, but would have little consequence for tethering to K-fibres, where it is more likely to rapidly re-bind a neighbouring MT, because of the high density of parallel MT bundles. This ‘kinetic proofreading mechanism’ (figure 2*a*) would thus ensure that CenpE transports the polar chromosomes towards the spindle equator along a preformed K-fibre of an already bioriented chromosome. Once CenpE has lost contact with Aurora A, PP1 removes the Thr422 phosphorylation and binds to CenpE to allow transport towards the midzone and enable end-on attachment by the kinetochore proteins Ndc80 and KNL1. One problem with this model is that it does not explain why both Aurora A and B are needed to phosphorylate CenpE at Thr422. The localization of Aurora A at the spindle poles brings it close to CenpE, which is loaded with laterally attached chromosomes. However, Aurora B is already in close proximity to CenpE, being enriched at the centromere. Aurora B-dependent Thr422 phosphorylation may allow reduction of CenpE processivity on incorrectly attached kinetochores in the metaphase plate, but this does not explain why Aurora A is needed to phosphorylate CenpE at laterally attached kinetochores at the poles. Possibly, an increase in Aurora kinase activity is needed at the poles to overcome PP1 activity and to displace the phosphatase from CenpE. It is also not clear whether CenpE is the only target through which Aurora A controls chromosome alignment, and to what extent Aurora A and B actually synergize in chromosome congression and biorientation. Double inactivation of Aurora A and B does not result in an obvious increase in alignment defects (figure 1*b*), arguing against a strong synergy. However, a more careful analysis of chromosome congression and biorientation in single and double Aurora A- and B-inactivated cells will be necessary to address this question.

### 5.2. Shared functions of Aurora kinases in the inhibition of microtubule depolymerases

Another function shared between Aurora A and B in the control of mitotic spindle dynamics is the inhibition of MT depolymerization in metaphase spindles. Kif18B and MCAK are two other kinesins that appear to be targets of Aurora A and B in this pathway [95]. The starting point of this discovery was an analysis of spindle morphology after Aurora A, Aurora B and Aurora A+B inactivation. Joint inactivation of both Auroras led to a dramatic loss of MTs. This could be reversed by co-depletion of the MT depolymerases Kif18B and MCAK, suggesting that these two enzymes are deregulated in the absence of Aurora kinase activity. The authors went on to show that Kif18B and MCAK directly interact at the plus end of MTs, guided by the MT plus-end-binding protein EB1. According to their model, Kif18B is required to transport MCAK along the MTs towards the very tip of the MT end (figure 2*b*). This is where MCAK acts as a depolymerase [98], although Kif18B may itself also contribute to MT depolymerization [99]. How Aurora kinases suppress MT depolymerization activity of these two enzymes remains unclear. Kif18B is a substrate for both Aurora A and B *in vitro*, but mutation of the identified phosphorylation sites did not have any effect on Kif18B localization and activity [95]. MCAK regulation by Aurora kinases is well documented, but its impact on MCAK function is complex [100]. There is a cluster of Aurora B kinase sites in the MCAK neck domain. Phosphorylation of S192 (S196 in *Xenopus*) is thought to reduce the affinity of the protein to MTs and does inhibit its MT depolymerization activity [66,67,101]. Moreover, N-terminal Aurora B phosphorylation sites in MCAK are thought to regulate its affinity to the centromere, but these can have both negative and positive impacts on centromere binding, suggesting a complex regulatory interplay between MCAK and Aurora B [66,67,101,102]. In fact, Aurora B and MCAK are supposed to work together in the correction of attachment errors by destabilizing synthetic attachments. Thus, differential effects of individual phosphorylation sites may allow specific modulation of MCAK activity by Aurora B with both negative and positive impacts, depending on the circumstance. Aurora A has also been reported to phosphorylate MCAK on S196, thereby further contributing to inhibition of depolymerase activity [62]. There is another Aurora site in the C-terminus that is required for localization of MCAK to the spindle poles and for Ran-dependent bipolar spindle formation in centrosomeless *Xenopus* egg extracts [62]. Moreover, Aurora A appears to be required for MCAK localization to centrosomes in mammalian cells [50]. Thus, Aurora A and B act as both positive and negative regulators of MCAK in a complex interplay of various phosphorylation sites.

Based on these data, we cannot as yet build a clear model to explain Tanenbaum *et al*.'s [95] observation that double inhibition of Aurora A and B causes a dramatic loss of spindle MTs owing to unchecked activation of MCAK and Kif18B. We need to determine which of these phosphorylation sites in Kif18B and/or MCAK are responsible for this effect, and how these sites contribute to MCAK/Kif18B complex formation and inhibition of MT depolymerization activity.

### 5.3. Shared functions of Aurora A and B in controlling anaphase microtubule depolymerization

Redundancy in CenpE and MCAK/Kif18B phosphorylation could be sufficient to explain the specific failure in chromosome segregation in cells lacking Aurora A and B. Alternatively, Aurora A and B could also collaborate in other aspects of mitotic MT dynamics. When studying Aurora function in DT40 cells, we discovered a synergistic effect of Aurora A and B inactivation in anaphase MT depolymerization resulting in a persistence of long spindle MTs following Cdk inactivation [2]. This was a truly synergistic effect and did not occur in cells lacking either Aurora A or B activity, but only after inactivation of both kinases. Rapid depolymerization of K-fibre MTs is thought to be a major contributor to force generation in spindle in anaphase that is required to pull the sister chromatids apart [9,103]. MT depolymerization can occur at both the MT plus and minus ends (figure 2*c*). Minus-end depolymerization causes the so-called flux movement of tubulin subunits along kinetochore fibres towards the spindle pole [104,105]. MT depolymerization at the plus end results in a release of energy from MT bending at the depolymerizing tip that could drag along the attached kinetochore [106]. The actual impact of MT Flux and/or Pacman on chromatid segregation is still under debate, and may vary among cell lines and organisms [105]. The respective localization of Aurora A and B at the centrosome (MT minus end) and kinetochore (MT plus end) points to a role for these kinases in plus- and minus-end MT depolymerization. The synergy in cells lacking both Aurora A and B could suggest that either minus- or plus-end depolymerization is sufficient to drive spindle disassembly and chromosome segregation in DT40 cells, and only inactivation of both pathways results in effective stabilization of anaphase MTs and failure in chromosome segregation. These hypotheses need to be addressed by measuring plus- and minus-end depolymerization directly in cells lacking Aurora A, B or both kinases.

The precise mechanisms of this regulatory pathway remain to be determined (figure 2*c*). One problem with this hypothesis is that Aurora B kinase as part of the CPC actually leaves the centromere and remains in the spindle midzone as the kinetochores are travelling with the shrinking K-fibres. The critical Aurora B-dependent phosphorylation event must therefore already happen in metaphase before the K-fibre MTs depolymerize. The real trigger of anaphase spindle disassembly is more likely to be a drop in Cdk activity, anaphase-promoting complex/cyclosome (APC/C)-mediated proteolysis and the activation of mitotic exit phosphatases. The impact of these factors on Aurora functions in MT dynamics remains to be determined. It also appears paradoxical that Aurora kinases should cooperate to stabilize the metaphase spindle and then be required for MT depolymerization in anaphase. A dual role of Aurora kinases as enhancers and suppressors of MT depolymerization already becomes apparent in the regulation of the MT depolymerase MCAK. Aurora kinase-dependent phosphorylation has both positive and negative effects on MCAK by localizing it to the right place while reducing its activity. The balance of this regulation may be changed in anaphase by the specific activation of phosphatases that remove the inhibitory phosphorylation, whereas the activating sites remain untouched. MCAK itself may, in fact, not be involved in anaphase K-fibre depolymerization. This function has been attributed to other members of the kinesin 13 family, namely Kif2a and Kif2b [107,108]. Aurora A appears to inhibit the MT depolmerase activity of Kif2a and also suppresses its binding to MTs, but not the spindle poles [63]. Regulation of Kif2b by Aurora kinases has, to our knowledge, not been reported. MT depolymerases are not the only conceivable targets of Aurora kinase to trigger anaphase MT depolymerization. MTs are stabilized by end-binding proteins, and in yeast, Ipl1 triggers spindle disassembly by phosphorylating Bim1, a member of the plus-end-binding proteins [77,98,109]. A minus-end-binding protein, patronin, has been recently discovered in *Drosophila* [110], but its mammalian homologues and its regulation by mitotic kinases remain unknown. A third class of proteins that play a role in anaphase MT dynamics are the MT-severing enzymes, including Katanin, Spastin and Fidgetin [111]. Studies in *Drosophila* have implicated these enzymes in triggering both plus- and minus-end destabilization of anaphase K-fibres [112]. Katanin appears to be negatively regulated by Aurora B in *X. laevis*, and this phosphorylation contributes to spindle size control [97]. A positive regulation of MT-severing enzymes by Aurora kinases has so far not been observed. Overall the changes in MT dynamics between metaphase and anaphase will require intensive investigation. These are likely to consist of a complex interplay of mitotic kinases to maintain positive regulatory phosphorylation. Moreover, local activation of specific phosphatases is also likely to play an important role to remove inhibitory phosphorylation sites that are required to maintain the steady state of metaphase MTs. Aurora kinases contribute to these phosphorylation events from both the minus and plus ends of the spindle MTs and coordinate the intricate regulatory network that controls mitotic spindle function.

## 6. Evolution of the Aurora kinase family

One possible way to learn about the functional split of Aurora kinases at the spindle pole and equator lies in studying the evolution of this kinase family. When considering Aurora evolution an immediate question arises: which form of Aurora constitutes the ancestral form? The fact that fungi only contain a single predominantly centromeric Aurora could suggest that this is the original orthologue, while Aurora A could have evolved later, in parallel with changes in the spindle pole structure and the occurrence of the centrosome. Alternatively, yeast could have lost the polar form of Aurora. However, even invertebrate and vertebrate Aurora kinases carry too little phylogenetic signal to conclude their ancestral relationship [113]. Moreover, Ipl1 has also been implicated in regulating spindle pole body cohesion in yeast meiosis [114], suggesting that this kinase may also act both on spindle poles and equator. Other organisms that lack centrosomes (such as plants) have several Aurora paralogues that localize to the spindle poles and the midzone [115]. There appear to be a variety of evolutionary routes different organisms have taken to relocalize Aurora kinase to both ends of the spindle MTs. *Dictyostelium* and starfish, for example, have only a single Aurora kinase that covers functions of both the polar Aurora A and the equatorial Aurora B [116,117].

**Figure 3.**
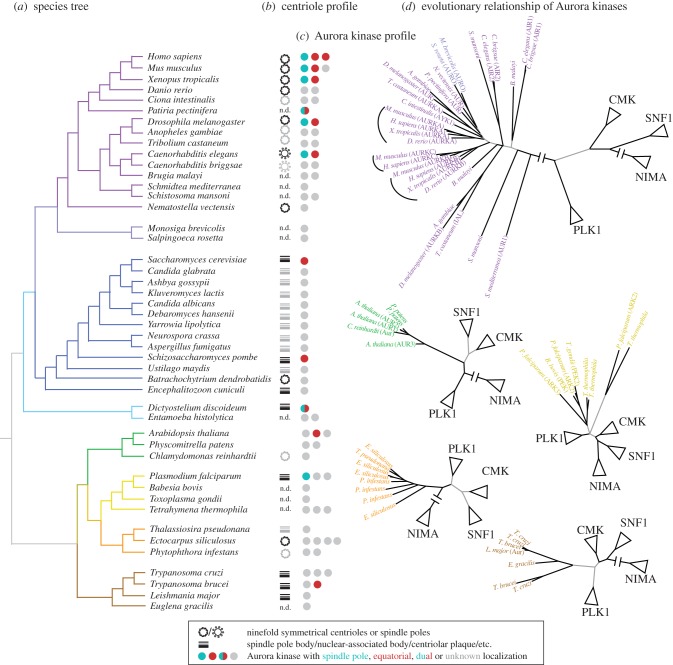
Aurora kinase evolution. (*a*) Eukaryotic tree [118–122]. (*b*) Presence and type of structure at the spindle pole [123,124]. Cylindrical structures (centrioles) exist in animals in the context of centrosomes, but they exist also in other organisms, albeit with unclear role in spindle formation. Additionally, many organisms have different structures at the spindle poles. (*c*) Number of Aurora kinases in the genome, identified via recursive BLAST searches from known Aurora/Ipl1 proteins, and classified as such by being monophyletic with Aurora/Ipl1 and not other related protein kinases. Red indicates an equatorial localization, green a spindle pole/centrosomal localization, and grey indicates that the localization of that kinase has not been described in the literature. Localization data were obtained from *H. sapiens* [19,25,125], *M. musculus* [126], *C. elegans* [21,127], *Xenopus* sp. [22,45,128], *P. pectinifera* [116], *S. cerevisiae* [129], *S. pombe* [130], *D. discoideum* [117], *A. thaliana* [115], *P. falciparum* [131], *T. brucei* [132]. (*d*) Dendograms (maximum likelihood) of Aurora kinases and related kinases for each evolutionary group except for fungi. Branches in grey have less than 60% bootstrap support.

To address these questions, we performed a systematic analysis of Aurora evolution by surveying the presence and conservation of the kinase family among eukaryotes and comparing this data with information on the structure of the spindle poles. Aurora kinases are found in all organisms that we investigated (figure 3), which suggests that its origin predates the radiation of eukaryotes, and that it was present in the last eukaryotic common ancestor (LECA). We found that every major eukaryotic group has at least one basal organism that has a single Aurora (figure 3*c*), suggesting that the ancestors of all these groups, and probably the LECA, possessed a single Aurora kinase gene. This is suggestive of multiple independent taxon-specific duplications giving rise to the extant constellation of Auroras, reaching three and four paralogues in some organisms. A phylogenetic analysis (figure 3*d*) further supports the notion that independent duplications have occurred in several branches, for example giving rise to Aurora C in mammals, or the multiple plant and moss Auroras. However, Aurora kinase sequences proved very resistant to phylogenetic analysis, a fact already noted by others [113], which makes the assignment of orthology relationships based on this method impossible over even very short evolutionary distances. This means that while the vertebrate signal is still clear and the monophyly of each group of Auroras is well established, this relationship is lost when invertebrates such as *Caenorhabditis elegans* or *Drosophila melanogaster* are included in the analysis (figure 3*d*). This method does not allow us to establish that Aurora in the fruitfly is the orthologue of the mammalian Aurora A, nor the nature of the evolutionary relationship between the well-studied animal kinases and their counterparts in other groups, including fungi. Thus, it is unclear which one may have been the ancestral Aurora function. It is also noteworthy that Aurora kinases have gained lineage-specific functions. Examples are their role in defining flagellar length in the *Chlamydomonas reinhardtii* [133], and the coupling of cytokinesis with kinetoplastid and nuclear division in *Trypanosoma brucei* [134–136]. These functional specializations are also reflected by the dramatic variation in the domain architectures of Auroras. For example, *C. reinhardtii* possesses a C-terminal extension, containing a MT-binding domain and a PEST motif [137].

Considering the sparse functional evidence currently available in non-model organisms (figure 3*c*), it appears that single paralogue Aurora kinases are either bifunctional or have solely an equatorial localization. This suggests that the equatorial B-type Aurora constitutes the ancestral form. This speculation is further supported by the fact that the structures at the poles of the spindle show considerable variation among different species, whereas the kinetochore is a common feature among eukaryotic chromosomes (figure 3*d*). In animals the spindle poles are associated with the centrosome and the Aurora A subfamily appears intimately linked to it. Accordingly, most organisms that have ninefold symmetrical centrioles do have both a polar and an equatorial Aurora, whereas species with centriole-less spindle pole bodies, such as yeast, generally have a single Aurora orthologue. One could thus hypothesize that the A-type Aurora kinase family coevolved with the centrosome. However, this is unlikely, because some organisms that contain centriole-less spindle pole bodies (such as *Dictyostelium* and *Plasmodium*) have a polar Aurora kinase. It will be important to analyse the Aurora kinase subcellular localization in species that contain centrosomes but only have a single Aurora kinase and in species that have centriole-less spindle pole bodies other than yeast. By comparing MT dynamics and chromosome segregation mechanism in organisms with and without polar Aurora activity, we may be able to determine why the polar version of Aurora kinase has evolved.

## 7. Conclusions and outlook

This review has emphasized a view on Aurora kinases focusing on concerted functions of the polar and equatorial forms of this kinase family. Even though the individual roles for A- and B-type Auroras are well studied, their combined functions in mitotic progression will need to be further elucidated. The molecular targets that lie beneath the synergistic effects on sister chromatid segregation and anaphase K-fibre depolymerization remain to be understood, and the list of overlapping substrates needs to be analysed for further evidence of functional redundancy. From an evolutionary perspective, a fascinating picture of a highly dynamic Aurora kinase family emerges. If equatorial B-type Aurora constitutes the ancestral form and polar Aurora A-type kinases have indeed evolved in parallel in different species both in the presence and absence of centrosomes, we really need to know what constitutes the selection pressure for this functional split. It will also be necessary to address why A-type Auroras are so divergent among relatively closely related phyla such as arthropods and vertebrates. If these kinases have indeed evolved separately, one could conclude that the evolutionary requirement for a separate polar Aurora has occurred relatively recently. Studying the conservation of overlapping and specific functions of Aurora A and B in different species will help to answer these questions. Given that large investments have been made to develop Aurora kinase inhibitors as cancer therapeutics, these questions gain an immediate urgency. The outcome of inhibiting Aurora A and B individually, or in combination on cellular ploidy, is clearly very different, and it will be important to determine how these different states of ploidy affect tumours of varying genetic make-up. It is conceivable that these differential consequences of specific inhibitors against each Aurora kinase, as well as pan-Aurora inhibitors, could be exploited separately in different cancer types, and could also provide potent synergies with other cancer drugs.
